# On the Molecular Pharmacology of Resveratrol on Oxidative Burst Inhibition in Professional Phagocytes

**DOI:** 10.1155/2014/706269

**Published:** 2014-01-28

**Authors:** Radomír Nosáľ, Katarína Drábiková, Viera Jančinová, Tomáš Perečko, Gabriela Ambrožová, Milan Číž, Antonín Lojek, Michaela Pekarová, Jan Šmidrkal, Juraj Harmatha

**Affiliations:** ^1^Institute of Experimental Pharmacology and Toxicology, Slovak Academy of Sciences, Dúbravská 9, 841 04 Bratislava, Slovakia; ^2^Institute of Biophysics AS CR v.v.i. Královopolská 135, 612 65 Brno, Czech Republic; ^3^Institute of Chemical Technology, Faculty of Food and Biochemical Technology, Technická 5, 166 28 Praha 6, Czech Republic; ^4^Institute of Organic Chemistry and Biochemistry AS CR v.v.i., Flemmingovo nám. 2, 166 10 Praha 6, Czech Republic

## Abstract

Resveratrol—3,5,4′-trihydroxystilbene—possesses antioxidant activities *in vitro*. It dose-dependently inhibited the generation of peroxyl, hydroxyl, peroxides, and lipid peroxidation products in cell free systems. Oxidative burst of whole human blood stimulated with PMA, fMLP, OpZ, and A23187 was inhibited in a concentration-dependent way, indicating suppression of both receptor and nonreceptor activated chemiluminescence by resveratrol. Results from isolated human neutrophils revealed that resveratrol was active extracellularly as well as intracellularly in inhibiting the generation of reactive oxygen species. Liberation of ATP and analysis of apoptosis showed that in the concentration of 100 **μ**M, resveratrol did not change the viability and integrity of isolated neutrophils. Western blot analysis documented that resveratrol in concentrations of 10 and 100 **μ**M significantly decreased PMA-induced phosphorylation of PKC **α**/**β**II. Dose-dependent inhibition of nitrite production and iNOS protein expression in RAW 264.7 cells indicated possible interference of resveratrol with reactive nitrogen radical generation in professional phagocytes. The results suggest that resveratrol represents an effective naturally occurring substance with potent pharmacological effect on oxidative burst of human neutrophils and nitric oxide production by macrophages. It should be further investigated for its pharmacological activity against oxidative stress in ischaemia reperfusion, inflammation, and other pathological conditions, particularly neoplasia.

## 1. Introduction

Neutrophils are present in high numbers in areas of inflammation, where they constitute an important source of reactive oxygen species (ROS). The massive production of antimicrobial and tumoricidal ROS in an inflammatory environment is called “oxidative burst” and plays an important role as the first line of defense against environmental pathogens. Paradoxically, however, neutrophils are also implicated in tissue-damaging inflammatory reactions that underlie the pathogenesis and exacerbation of many inflammatory diseases [1, 2]. There are at least two signalling pathways responsible for induction of neutrophil activation: one is the protein kinase C (PKC) mediated pathway, which can be driven by stimulation with phorbol-4*β*-12*β*-myristate-13*α*-acetate (PMA), and the other is the *Src* family protein tyrosine kinase mediated pathway [3].

Apoptosis is critical for the regulation of life span of circulating as well as emigrated neutrophils. Accumulating evidence indicates that neutrophil apoptosis is one of the critical determinants of the outcome of the inflammatory response and is a potential target for therapeutic interventions. A delay of neutrophil apoptosis exacerbates and prolongs inflammation or even prevents spontaneous resolution of inflammation [4]. The apoptotic neutrophil and the process of cell death exert anti-inflammatory effects that have been shown to be of therapeutic value in inflammatory diseases [5, 6].

A wide array of phenolic substances, particularly those present in edible and medicinal plants, have been reported to possess substantial antioxidative, anticarcinogenic, and antimutagenic activities by modulating important cellular signalling processes [7–10]. Natural polyphenols suppressed oxidative burst of stimulated human neutrophils by enhancing their apoptosis and decreasing protein kinase C activation [11–17].

Resveratrol (RES), a polyphenolic phytoalexin, is one of the most extensively studied natural products, with wide-ranging biological activities and tremendous clinical potential [18]. RES has been shown to have antioxidant, antiinflammatory, antiproliferative, and anti-angiogenic effects, while those on oxidative stress are presumably the most important [19].

In spite of the fact that almost 5000 papers are evidenced in PubMed database, there is a lack of evidence about the mechanism of the effect of resveratrol on oxidative burst in human professional phagocytes at molecular level. In this study, we investigated the effect of RES on the mechanism of oxidative burst in human whole blood, isolated neutrophils at extra- and intracellular level, activation of protein kinase C, caspase-3 activity and cellular viability and on free radical scavenging activity in cell free systems (oxygen radical absorption capacity—ORAC, hydroxyl radical averting capacity—HORAC, scavenging of ROS generation, nitric oxide production, and inhibition of lipid peroxidation). Moreover, we studied the effect of resveratrol on nitrite production and iNOS protein expression in murine RAW 264.7 macrophage cell line.

## 2. Methods and Materials 

### 2.1. Chemicals

Luminol, isoluminol, PMA (phorbol-4*β*-12*β*-myristate-13*α*-acetate), Ca^2+^-ionophore A23187, superoxide dismutase, dextran (average MW 464,000), zymosan A (from *Saccharomyces cerevisiae*), luciferase (from firefly* Photinus pyralis*), and D-luciferin sodium salt from Sigma-Aldrich Chemie (Deisenhofen, Germany). HRP (horseradish peroxidase), catalase, and Folin-Ciocalteu's phenol reagent were purchased from Merck (Darmstadt, Germany) and Lymphoprep (density 1.077 g/mL) from Nycomed Pharma AS (Oslo Norway). 2,2′-Azobis(2-methylpropionamidine) dihydrochloride (AAPH), 6-hydroxy-2,5,7,8-tetramethylchroman-2-carboxylic acid (Trolox), fluorescein disodium salt, cobalt(II) fluoride tetrahydrate, and gallic acid were obtained from Sigma-Aldrich (Steinheim, Germany). Picolinic acid was purchased from Fluka (Deisenhofen, Germany), and human purified caspase-3 was from Enzo Life Sciences, Lausen, Switzerland. All other chemicals used were of analytical grade and obtained from commercial sources.

ORAC and HORAC analyses were carried out on a FLUOstar Galaxy plate reader (BMG Labtechnology, Offenburg, Germany).

The phosphate buffered saline solution (PBS) used in this study contained 136.9 mM NaCl, 2.7 mM KCl, 8.1 mM Na_2_HPO_4_, 1.5 mM KH_2_PO_4_, 1.8 mM CaCl_2_, and 0.5 mM MgCl_2_  × 6H_2_O and had a pH of 7.4. Tyrode's solution used in this study consisted of 136.9 mM NaCl, 2.7 mM KCl, 11.9 mM NaH_2_CO_3_, 0.4 mM NaH_2_PO_4_  × 2H_2_O, 1 mM MgCl_2_  × 6H_2_O, and 5.6 mM glucose, pH of 7.4.

Resveratrol (RES) was prepared by targeted regioselective synthesis purely as transisomer [20] and was diluted in 1 : 50 (v/v) of 1 M NaOH in water.

### 2.2. Blood Collection and Neutrophil Separation

Fresh human blood was obtained at the blood bank by venipuncture from healthy male volunteers (20–50 years) who had not received any medication for at least 7 days. It was anticoagulated with 3.8% trisodium citrate (blood : citrate ratio = 9 : 1). The Ethical Committee license for blood sampling at the National Transfusion Service NTS-KRA/2012/SVI was registered. Human neutrophils were isolated from whole blood, as described previously [21, 22]. The final suspension of neutrophils contained more than 96% of viable cells, as evaluated by trypan blue exclusion and was used within 2 h, as long as the control chemiluminescence remained constant.

### 2.3. Chemiluminescence (CL) Assay of Whole Blood and Isolated Neutrophils

The oxidative burst in whole blood was stimulated with phorbol myristate acetate (PMA 0.05 *μ*M), opsonized zymosan (OpZ; 0.5 mg/mL), fMLP (1 *μ*M), or Ca ionophore A23187 (1 *μ*M). CL was measured in 250 *μ*L samples consisting of 50 *μ*L aliquots that contained blood (50× diluted), luminol (200 *μ*M), RES (0.01–100 *μ*M), and phosphate buffer [11]. The effect of RES on extra- and intracellular ROS production was measured in unstimulated and PMA (0.05 *μ*M) stimulated neutrophils (5 × 10^5^ per sample) by isoluminol/luminol-enhanced CL. The CL of both whole blood and isolated neutrophils was evaluated and measured in a microplate luminometer Immunotech LM-01T (Czech Republic) at 37°C [23].

### 2.4. Analysis of Apoptosis

Citrated whole human blood was collected as described above. Dextran (3%) was added (blood : dextran = 2 : 1) and centrifuged at 10 ×g at room temperature [16]. Before use, 1 mL of buffy coat that contained leukocytes was collected and stored on ice. The cells were counted on the hemocytometer (Coulter Counter), which focused on granulocytes. The cell suspension was adjusted to get 2 × 10^5^ neutrophils per sample. Three different concentrations of RES (1, 10, and 100 *μ*M) were applied and incubated with a control sample at 37°C for 10 min. The cells were stained with Annexin V, conjugated with FITC (BenderMedSystems) in the dark at 4°C for 10 min, followed by staining with propidium iodide (1 *μ*g/mL), and then analysed immediately by the Beckman Coulter Cytomics FC500 cytometer (for details, see Perečko et al. [16]).

### 2.5. Neutrophil Integrity

The cytotoxic effect of RES was evaluated by means of ATP liberation by luciferin-luciferase chemiluminescence [11]. The neutrophil suspension (30 *μ*L; 30 000 cells/sample) and 20 *μ*L of Tyrode's solution were incubated with 50 *μ*L of RES (1 to 100 *μ*M) for 15 min at 37°C. The total ATP content was assessed immediately after sonication of neutrophils for 10 s.

### 2.6. Recombinant Caspase-3 Activity

To determine the caspase-3 activity, a modified method was applied [16]. The final reaction with luciferase was detected by CL. The light production was measured in the Luminometer Immunotech LM-01T. The reagent was added and the mixture was measured for 60 min to determine caspase-3 activity. The solvent for RES, containing NaOH, was also evaluated.

### 2.7. Protein Kinase C Activation

Phosphorylation of protein kinase (PKC) isoenzymes *α* and *β*II was detected [11]. Isolated human neutrophils (5 × 10^6^) were incubated at 37°C with RES for 1 min, stimulated with PMA (0.15 *μ*M, 1 min), and lysed by the addition of solubilisation buffer. After sonication on ice, samples were centrifuged to remove unbroken cells, the supernatant was boiled for 5 min with sample buffer, and samples were loaded on 9.8% SDS polyacrylamide gels. Membrane strips were blocked for 60 min with 1% bovine serum albumin in Tris buffered saline. This was followed by 60 min incubation in the presence of the phospho-PKC *α* and *β*II (Thr638/641) antibody (rabbit anti-human, 1 : 8000, Cell Signaling Technology) or *β*-actin antibody (rabbit anti-human, 1 : 4000, Cell Signaling Technology, Danvers, MA, USA). The membranes were subsequently washed six times with TBS and incubated 60 min with the secondary antibody conjugated to horseradish peroxidase (anti-rabbit from donkey, 1 : 10,000, Amersham, UK). The optical density of each PKC band was corrected by the optical density of the corresponding *β*-actin band.

### 2.8. Cell Culture

Murine peritoneal macrophage cell line RAW 264.7 (American Type Culture Collection, USA) was cultivated in Dulbecco's Modified Eagle Medium (PAN, Germany) supplemented with 10% foetal bovine serum (PAN, Germany) and 1% gentamycin (Sigma, USA). Cells were maintained at 37°C and 5% CO_2_. After reaching confluence, cells were harvested and washed. Cell numbers and viability were determined by ATP test [24].

### 2.9. Formation of Nitric Oxide

Generation of reactive nitrogen species was determined indirectly as the accumulation of nitrites in the supernatant of murine macrophages RAW 264.7 [12]. Control cells were incubated with LPS without RES treatment. At the end of the incubation period, culture media were collected from wells and centrifuged at 5000 ×g and 4°C for 5 min. Then 150 *μ*L of supernatant was mixed with equal volume of Griess reagent (Sigma, USA) in a 96-well plate, and the mixture was incubated at room temperature and in the dark for 30 min. The cell fractions of these samples were used for the detection of inducible NO synthase expression by Western blot.

### 2.10. Western Blot Analysis of iNOS Expression

After removing the supernatant for nitrite measurement, the remaining cells were washed with cold phosphate buffered saline (PBS) and lysed in the lysis buffer (1% sodium dodecyl sulphate—SDS, 10^−1^ M Tris pH 7.4, 10% glycerol, 10^−3^ M sodium ortho-vanadate, 10^−3^ M phenylmethanesulfonyl fluoride). Protein concentrations were determined by using BCATM protein assay (Pierce, USA), with bovine serum albumin as standard. Equal amounts of protein were then subjected to SDS-polyacrylamide gel electrophoresis using 7.5% running gel. The expression of iNOS protein was quantified by Western blot analysis [25]. Relative protein levels were quantified by scanning densitometry using the Image JTM programme, and the individual band density value was expressed in arbitrary units.

### 2.11. Antioxidant Activity Assays (Free Radical Scavenging Activity in Cell Free Systems)

#### 2.11.1. ORAC Assay

The ORAC method measures the antioxidant scavenging activity against peroxyl radical induced by 2,2′-Azobis(2-methylpropionamidine) dihydrochloride (AAPH) at 37°C [26, 27]. Fluorescein (FL) was used as the fluorescent probe. The protective effect of an antioxidant was measured by assessing the area under the fluorescence decay curve (AUC). The final ORAC values were calculated using a regression equation between the Trolox concentration and the net area under the curve.

#### 2.11.2. HORAC Assay

HORAC measures the metal-chelating activity of antioxidants under the conditions of Fenton-like reactions employing a Co(II) complex and hence the protecting ability against formation of hydroxyl radical [26, 27]. The initial fluorescence was measured, after which the readings were taken every minute after shaking in the presence of 100, 200, 400, 500, and 600 *μ*M gallic acid solutions (in phosphate buffer 75 mM, pH 7.4). The final HORAC values were calculated using a regression equation between gallic acid concentration and the net area under the curve.

### 2.12. ROS Scavenging in Luminol-Horseradish Peroxidase (HRP)-H_2_O_2_ Cell Free System

Aliquots of 50 *μ*L of RES solutions, HRP (2 U/mL), and luminol (10 *μ*M) were mixed in a 96-well luminescence plate to yield final concentrations of RES 1, 10, and 100 *μ*M. The reaction was started by adding hydrogen peroxide at the final concentration of 100 *μ*M (final volume of the sample was 200 *μ*L). Chemiluminescence was measured for 10 minutes at 37°C with Luminometer Immunotech LM-01T (Beckman Coulter).

### 2.13. NO Scavenging Activity

The potential ability of extracts to scavenge NO in chemical systems was tested by electrochemical measurement of NO [28]. NO was measured using three electrode systems: a porphyrinic microsensor working electrode, counter electrode, and a reference electrode were connected to the ISO-NO MARK II potentiostat (WPI, USA) [28]. The injection of the 2 *μ*L NO-saturated water into the measurement glass vial (final concentration of NO = 2.38 *μ*M) caused a rapid increase with subsequent gradual decrease of the NO induced signal until it reached the background current [29]. The scavenging properties of the extracts tested were evaluated as the time needed for reaching again the background current.

### 2.14. Lipid Peroxidation

The amount of 0.9 mL of 0.5 mM *α*-linolenic acid (Sigma-Aldrich, Steinheim, Germany) was mixed with 0.1 mL sample. Then, a system generating hydroxyl radical (0.1 mL Co(II) and 0.1 mL hydrogen peroxide for details, see section HORAC) was added for the induction of lipid peroxidation and the mixture was incubated for 2 h in 37°C. The concentration of thiobarbituric acid-reactive substances (TBARS) was measured as the index of lipid peroxidation [30]. The absorbance of the upper layer was measured at 532 nm. 1,1,3,3-Tetraethoxypropane (Sigma-Aldrich, Steinheim, Germany) in the final concentration of 0.1 *μ*M was used as standard. Lipid peroxidation was expressed in nM of TBARS per 1 mL of the mixture *α*-linolenic acid/analysed sample.

### 2.15. Statistical Analysis

Data represent the mean ± SEM, unless stated otherwise. Statistical analysis was performed using the ANOVA paired test to examine differences between the treatments and control. Differences were considered to be statistically significant when *P* < 0.05 (*) or *P* < 0.01 (**).

## 3. Results

**Figure 1:**
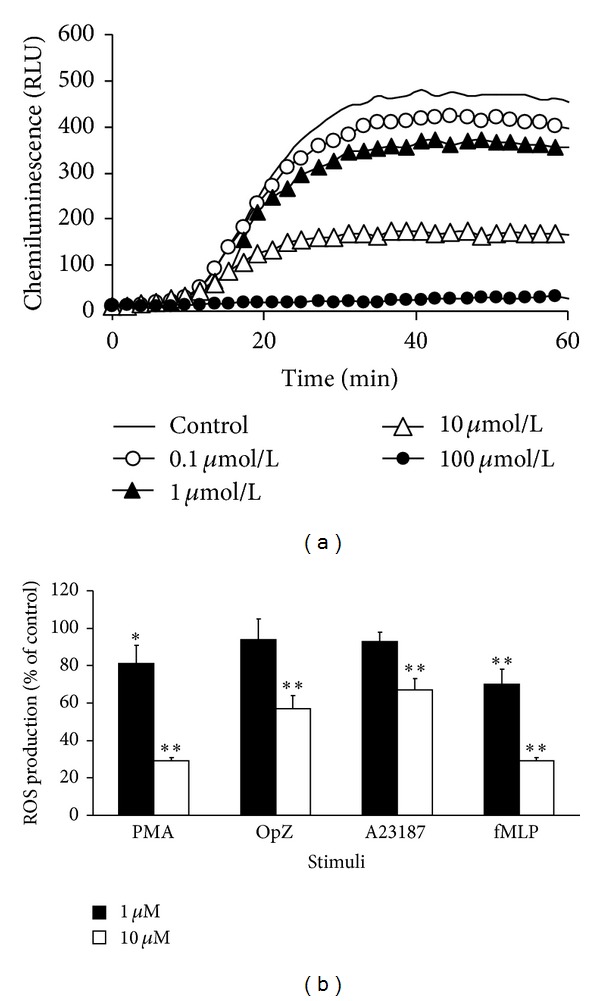
(a) Resveratrol dose-dependently decreased luminol-enhanced representative chemiluminescence curves of human whole blood stimulated with phorbol myristate acetate (PMA = 0.05 *μ*M) at 37°C. (b) Effect of resveratrol in 1 and 10 *μ*M concentration on PMA (0.05 *μ*M), OpZ (0.5 mg/mL), fMLP (1 *μ*M), and A23187 (1 *μ*M) stimulated chemiluminescence. *n* = 6–8, Mean ± SEM, *P ≤ 0.05; **P ≤ 0.01.

Figure 1(a) demonstrates representative dose-dependent CL curves of whole blood treated with RES and stimulated with PMA (0.05 *μ*M).  Figure 1(b) shows the effect of RES in 1 and 10 *μ*M concentration on whole human blood CL stimulated with PMA (0.05 *μ*M), OpZ (0.5 mg/mL), A23187 (1 *μ*M), and fMLP (1 *μ*M). In 1 *μ*M concentration, RES significantly decreased CL for PMA and fMLP stimuli to 19 and 30 per cent of control value (=100%), respectively. RES in 10 *μ*M concentration significantly inhibited CL with all stimuli applied in the rank order of potency PMA = fMLP > OPZ > A23187 demonstrating an evident difference in resveratrol activity to decrease stimulated chemiluminescence of whole blood.

**Figure 2:**
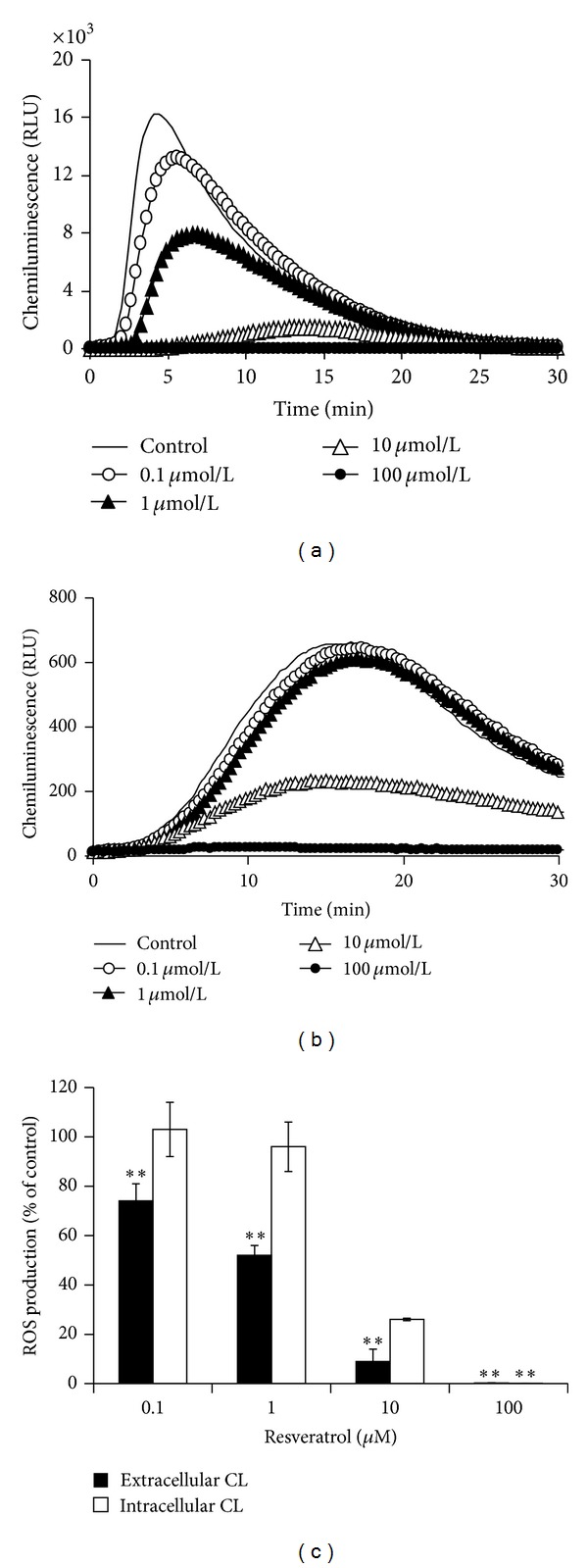
Chemiluminescence of isolated neutrophils. (a) Dose-dependent extracellular representative chemiluminescence (luminol + peroxidase) curves of human isolated neutrophils pretreated with resveratrol and stimulated with PMA (0.05 *μ*M). (b) Intracellular dose-dependent representative chemiluminescence (isoluminol + catalase + superoxide dismutase) curves of isolated human neutrophils pretreated with resveratrol and stimulated with PMA (0.05 *μ*M). (c) Dose-dependent effect of resveratrol on PMA (0.05 *μ*M) stimulated extracellular and intracellular chemiluminescence. *n* = 6–8; mean ± SEM, **P ≤ 0.01.

The effect of RES on extra- and intracellular CL of isolated neutrophils stimulated with PMA is demonstrated in Figure 2. It is evident that RES dose-dependently decreased extracellular CL, significantly starting at 0.1 *μ*M concentration. At 100 *μ*M concentration (Figure 2(a)), there was complete inhibition of stimulated CL due to PMA. At intracellular level (Figure 2(b)), RES significantly decreased CL at 10 and 100 *μ*M concentrations to 26 and 0.33 percent of the control value, respectively (Figure 2(c)).

Isolated intact neutrophils liberate 18 ± 3.8 nM ATP, which represents 3.2% from the total ATP amount (548 ± 112 nM/3 × 10^4^ cells). RES in any concentration used did not liberate ATP from isolated neutrophils (results not shown), indicating that RES did not disintegrate isolated neutrophils in any concentration used.

Table 1 demonstrates the effect of RES in concentrations of 1 to 100 *μ*M on viability of isolated neutrophils. In concentrations of 1 and 10 *μ*M, RES did not change significantly the amount of dead cells as compared with control cells. In 100 *μ*M, concentration RES increased the number of apoptotic cells from 7.9% (controls) to 18.6% of the total viable cells. The number of dead cells did not increase. These results show that RES in the optimal concentrations used (1 and 10 *μ*M) did not change apoptosis of isolated human neutrophils.

**Figure 3:**
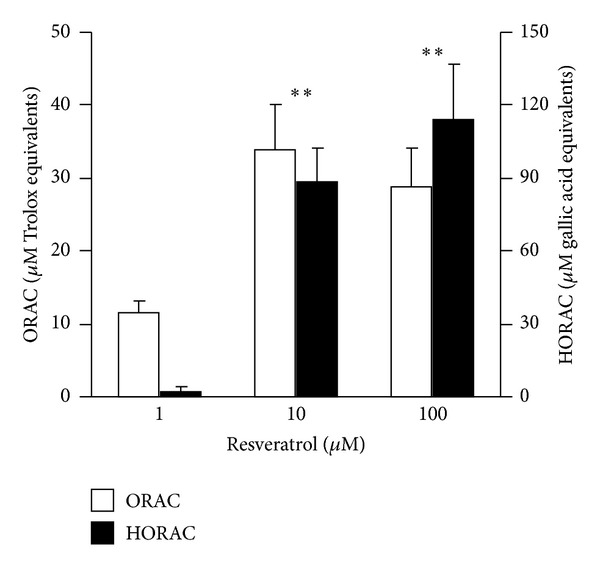
Effect of resveratrol on hydroxyl (HORAC) and peroxyl (ORAC) radical scavenging activity in cell free system expressed as gallic acid and Trolox equivalents, *n* = 4, mean ± SEM, **P ≥ 0.01.

Figure 3 shows the result of RES on radical scavenging activity in cell free system. RES in concentrations 10 and 100 *μ*M increased hydroxyl scavenging activity (HORAC) to 88 and 114 *μ*M of gallic acid equivalents, respectively, and peroxyl scavenging activity (ORAC) to 34 and 29 of Trolox equivalents, respectively. This effect demonstrates effective scavenging activity of RES on hydroxyl and peroxyl radicals *in vitro.*

**Figure 4:**
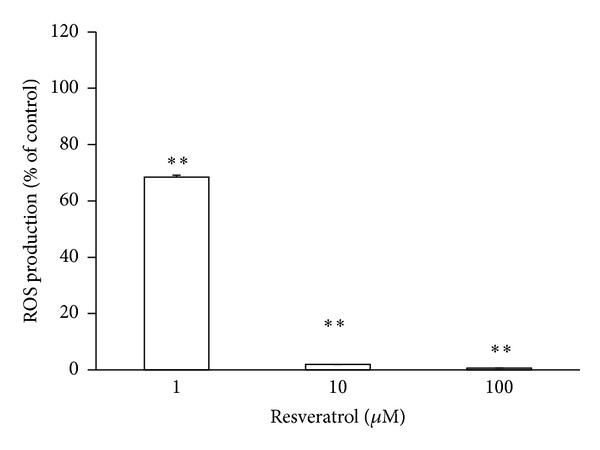
Dose-dependent effect of resveratrol on reactive oxygen species generated in cell free system by means of luminol + hydrogen peroxide + horseradish peroxidase. *n* = 3, mean ± SEM, **P ≥ 0.01.

Figure 4 shows the inhibitory effects of different concentrations (1, 10, and 100 *μ*M) of RES on production of ROS in cell free system generated by means of luminol + hydrogen peroxide + HRP. RES in 1 *μ*mol/L concentration significantly (*P* < 0.01) inhibited and in 10 *μ*M concentration totally blocked the chemiluminescence of 100 *μ*M hydrogen peroxide in the samples.

**Figure 5:**
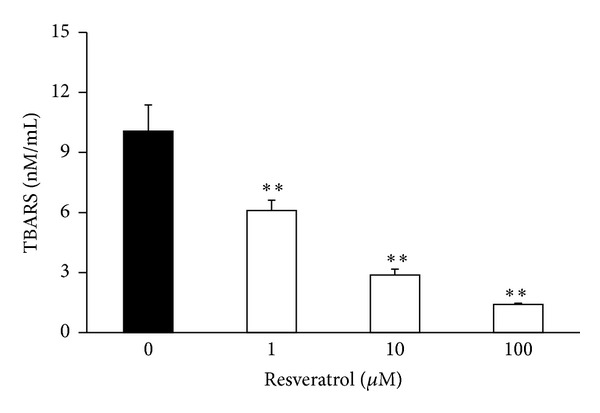
Effect of resveratrol on lipid peroxidation of *α*-linolenic acid expressed as thiobarbituric acid-reactive substances (TBARS), induced by hydroxyl radicals. *n* = 6, mean ± SEM, **P ≥ 0.01.

Since lipids are very susceptible to lipid peroxidation, we tested also the ability of RES to prevent the peroxidation of polyunsaturated fatty acids induced by hydroxyl radical. It is evident from Figure 5 that RES in all tested concentrations significantly and dose-dependently inhibited lipid peroxidation.

**Figure 6:**
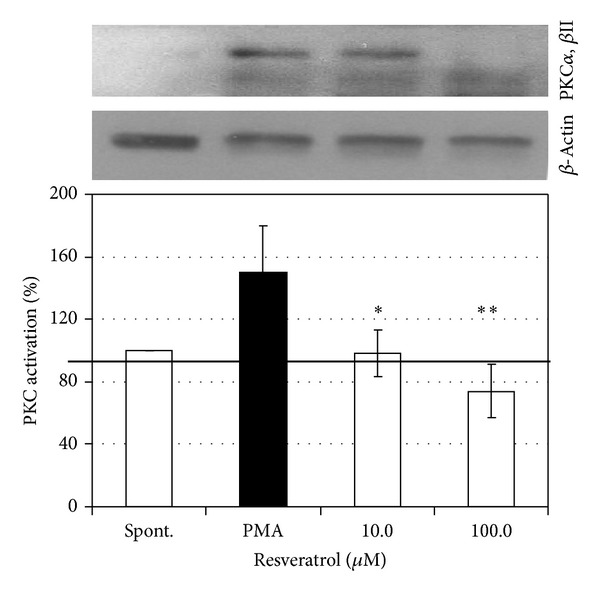
Western blotting analysis of protein kinase C activation in isolated human neutrophils pretreated with resveratrol (10 and 100 *μ*M) and stimulated with PMA (0.15**** *μ*M). *n* = 3-4, mean ± SEM; *P ≤ 0.05.

The activation of protein kinase C in isolated neutrophils stimulated with PMA (0.15 *μ*M) in the presence of 10 and 100 *μ*M RES is demonstrated in Figure 6. In both concentrations applied, RES reversed PMA stimulated PKC activation to spontaneous (control) values indicating a suppressive effect of RES on the activity of protein kinase C, one of the essential regulatory enzymes in reactive oxygen generation.

**Figure 7:**
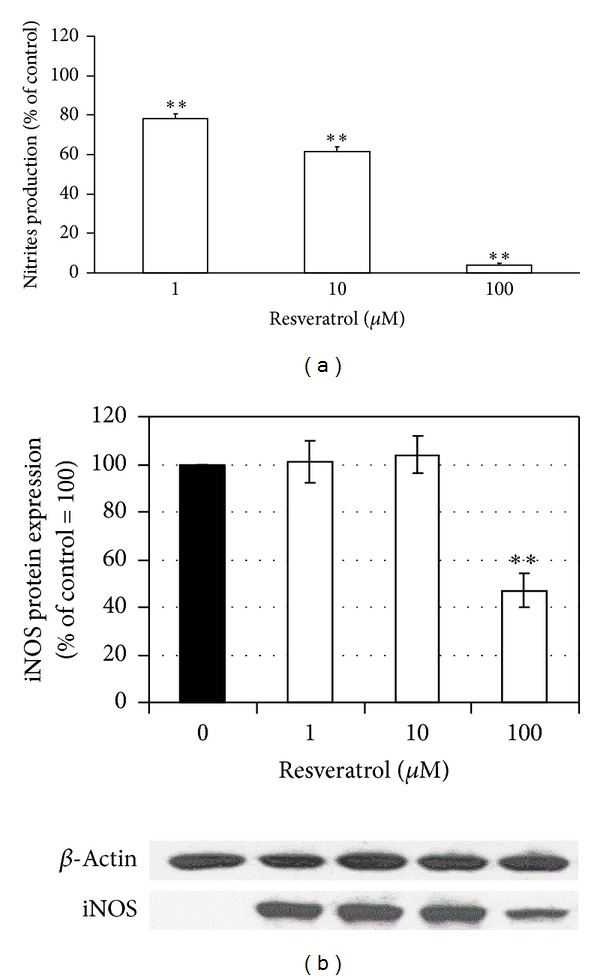
Nitrite production and iNOS expression in RAW 264.7 culture cells. Nitrite production (a) and densitometric analysis together with representative Western blot of iNOS protein expression (b) in LPS stimulated RAW 264.7 cells treated with resveratrol. *n* = 3, mean ± SEM, **P < 0.01.

The effect of RES on nitrite production and iNOS expression in RAW 264.7 cell culture after LPS activation is demonstrated in Figure 7.  Figure 7(a) demonstrates that RES in concentrations of 1, 10, and 100 M decreased nitrite concentration in cell supernatants to 82, 65, and 6 percent of the control value, respectively.

Figure 7(b) shows the effect of RES on iNOS expression determined by Western blot analysis. In comparison with the iNOS protein level in the control sample, iNOS protein expression was significantly inhibited only by the highest concentration (100 *μ*M) of RES to 60% of the control value.

## 4. Discussion

Resveratrol dose-dependently inhibited oxidative burst in human whole blood stimulated with two membrane-bypassing (PMA, A23187) and two membrane-operating stimuli (OpZ, fMLP). There was no significant difference between the stimuli applied and chemiluminescence decrease of whole blood indicating that RES may not act only as an extracellular scavenger but suppresses oxidative burst also intracellularly. This suggestion was confirmed on isolated neutrophils (Figure 2) stimulated with PMA demonstrating that RES in a concentration dependent way inhibited not only extracellularly determined chemiluminescence but effectively suppressed formation of intracellularly generated ROS (Figure 2(b)). The difference was evident; RES started to inhibit extracellular chemiluminescence at 0.1 *μ*M, intracellularly at 10 *μ*M concentration (Figure 2(c)).

The stimulated generation of ROS in whole blood and isolated neutrophils was decreased by many polyphenolic compounds like curcumin, pterostilbene, pinosylvin, and N-feruloyl serotonin [11, 14, 31]. Inhibition of fMLP-activated human neutrophil chemiluminescence was accompanied by inhibition of elastase and *β*-glucuronidase secretion and production of 5-lipoxygenase metabolites leukotriene B4, 6-trans-LTB4, and 12-trans-epi-LTB4 after stimulation with calcium ionophore, indicating that transresveratrol interferes with the release of inflammatory mediators in activated polymorphonuclear leukocytes [32].

Since RES did not liberate ATP from isolated neutrophils, it is evident that even in higher concentrations used (up to 100 *μ*M) there was no disintegration of cells. Moreover, RES dose-dependently decreased spontaneous ATP liberation.

RES did not change significantly the number of dead cells even in the highest concentration used, decreasing the number of live cells by 10.7% (Table 1). In contrast, RES decreased the activity of recombinant caspase-3 activity in cell free system, significantly at 10 *μ*M concentration. This result has to be verified in the cellular model since apoptosis, a programmed cell death, appears to be the most frequent fate of cells treated with RES [33]. Moreover, RES induced autophagy in human U251 glioma cells [34], decreased the intracellular reactive oxygen species level, which correlated with the induction of caspase-8 and caspase-3 cleavage in human colon cancer cells [35] and induced apoptosis in patients with chronic myeloid leukemia cells [36].

The antioxidant properties of RES were analysed via five different methods: ORAC(peroxyl), HORAC(hydroxyl), hydrogen peroxide-peroxidase dependent chemiluminescence, NO scavenging, and lipid peroxidation inhibition. The chosen methods embrace different aspects of the antioxidant action and give a comprehensive view on the antioxidant potential of the sample investigated.

In the following experiments, we tested also the scavenging properties of RES against NO, using electrochemical analysis which is considered to be a reliable method for verifying NO scavenging. However, no scavenging properties of resveratrol against NO were found in any concentration used.

These observations confirmed previous findings that RES is both a free radical scavenger and a potent antioxidant because of its ability to promote the activities of a variety of antioxidant enzymes [37]. The result of decreasing dose-dependently LDL oxidation in cell free system is supportive of its effect on lipid peroxidation and atherosclerotic lesion formation in animal hypercholesterolemic models [38].

RES decreased protein kinase C activation in PMA-stimulated neutrophils, indicating its interference with oxidative burst in neutrophils. Similar results were demonstrated in human gastric adenocarcinoma and CaSki cells [39, 40] and for the polyphenolic compound N-feruloyl serotonin in human neutrophils [14]. By inhibiting the activation of PKC [41], RES may interfere with modulation of intracellular signalling pathways involved in downregulation of COX-2 and iNOS expression and NF-*κ*B activation [10, 42]. Nitric oxide, a member of reactive nitrogen species, is an important molecule involved in the regulation of many physiological and microbicidal processes. RES markedly inhibited NO production by LPS stimulated macrophages. This finding corresponds with the latest results of other authors [43, 44] who also reported suppression of inducible nitric oxide synthase expression and NO production in macrophages after RES administration. Our results showed that resveratrol reduced nitrite accumulation more effectively than it reduced iNOS protein expression in stimulated macrophages through a mechanism which is at least partially independent of the regulation of iNOS protein expression. Nevertheless, in electrochemical measurements we showed that RES was not able to scavenge NO, suggesting that the direct scavenging activity against NO resulting from the inhibitory action of RES can be excluded.

In conclusion, RES possesses antioxidant activities *in vitro* inhibiting generation of ROS in cell free systems, oxidative burst of stimulated blood and isolated neutrophils both at extracellular and intracellular level, as measured by chemiluminescence.

Oxidative burst inhibition of human blood and isolated neutrophils, suppression of free radical generation and NO formation in cell free system confirmed the antioxidative properties and supported the effort to enlarge clinical studies with RES.

## Figures and Tables

**Table 1:** Effect of resveratrol in concentrations of 1 to 100 *μ*M on viability of isolated neutrophils. Cells were incubated with resveratrol at 37°C for 10 min, stained with Annexin V, subsequently conjugated with FITC in the dark at 4°C for 10 min, followed by staining with propidium iodide (1 *μ*g/mL), and then analysed immediately by the Beckman Coulter Cy. *n* = 4–6, mean ± SEM.

Resveratrol (*µ*M)	Live cells	Apoptotic cells	Dead cells
0	91.90 ± 1.02	7.90 ± 1.00	0.20 ± 0.04

1	92.20 ± 1.31	7.60 ± 1.30	0.20 ± 0.05
10	92.10 ± 1.16	7.80 ± 1.60	0.10 ± 0.03
100	81.20 ± 2.73**	18.60 ± 2.70**	0.20 ± 0.03

**P ≤ 0.01.
